# Therapeutic drug monitoring of voriconazole and the impact of inflammation on plasma trough concentrations in children

**DOI:** 10.3389/fphar.2025.1575233

**Published:** 2025-06-13

**Authors:** Lin Hu, Changyu Wang, Xi Tang, Qi Huang, Yanfei Li, Shiqiong Huang

**Affiliations:** ^1^ Department of Pharmacy, The Affiliated Changsha Hospital of Xiangya School of Medicine, Central South University, Changsha, Hunan, China; ^2^ Department of Pharmacy, The First Hospital of Changsha, Changsha, Hunan, China; ^3^ School of Mathematics and Physics, Wenzhou University, Wenzhou, Fujian, China; ^4^ Department of Pharmacy, Xiangya Hospital, Central South University, Changsha, Hunan, China

**Keywords:** voriconazole, inflammation, C-reactive protein, therapeutic drug monitoring, children

## Abstract

The aim of this study was to investigate the factors influencing voriconazole (VRC) plasma trough concentrations (*C*_trough_) in children and to provide a scientific basis for individualized VRC dosing. A retrospective study was conducted on children aged ≤18 years who received VRC treatment between 1 December 2017, and 31 December 2022. Medical data were collected to examine the relationship between VRC *C*_trough_ and non-genetic factors. A total of 59 patients were included in the study, with 90 VRC *C*_trough_ analyzed. The median patient age was 13 years (range, 1–18 years), and the median weight was 37.9 kg (range, 10.0–77.7 kg). The median number of VRC *C*_trough_ measurements per patient was 1 (range, 1–10). Inflammation, as indicated by C-reactive protein (CRP) levels, was significantly associated with dose-adjusted VRC *C*_trough_ (*C*_trough_/D) (n = 90, r = 0.746, *P* < 0.001). Patients with severe inflammation had significantly higher VRC *C*_trough_/D compared to those with mild inflammation (*P* = 0.001). The proportion of supratherapeutic concentrations was highest in the severe inflammation group, significantly higher than in the mild inflammation group (41.7% vs. 11.9%; *P* = 0.037). A significant correlation was found between VRC *C*_trough_/D and CRP concentrations in patients aged ≥12 years (n = 54, r = 0.784, *P* < 0.001), but no correlation was observed in patients aged <12 years (n = 36, r = 0.199, *P* = 0.244). A linear mixed model demonstrated a significant association between VRC *C*_trough_/D and CRP (β = 0.448; 95% CI, 0.309–0.587). Additionally, total bilirubin (TBil) (*P* = 0.039), direct bilirubin (DBil) (*P* = 0.034), albumin (ALB) (*P* = 0.011), and serum creatinine (Scr) (*P* = 0.008) were significantly associated with VRC *C*_trough_/D. These findings indicate that CRP levels should be considered a key factor influencing VRC exposure in pediatric patients. The relationship between VRC *C*_trough_ and CRP levels varies across age groups and should be analyzed separately.

## Introduction

Invasive aspergillosis has become a leading cause of death in patients with severe immune dysfunction, particularly among individuals with acute leukemia and those undergoing hematopoietic stem cell transplantation (Giannella et al., 2024). Voriconazole (VRC), a broad-spectrum triazole antifungal agent, is recommended as the first-line treatment for invasive aspergillosis in children according to clinical guidelines (Warris et al., 2019).

Given its complex pharmacokinetic profile, therapeutic drug monitoring (TDM) of VRC is crucial for optimizing treatment in pediatric patients. VRC plasma trough concentrations (*C*_trough_) exhibit significant inter- and intra-individual variability in children and adolescents, influenced by various factors. Our previous studies have identified genetic factors, such as CYP2C19 polymorphisms, as key contributors to this variability (Hu et al., 2023a; Hu et al., 2023b). Non-genetic factors also contribute to VRC *C*_trough_ variability. For instance, Zhao et al. (Zhao et al., 2021) reported that age, weight, dose, direct bilirubin (DBil), and blood urea nitrogen (BUN) were significant factors of VRC *C*_trough_. Similarly, Liu et al. (Liu et al., 2017) observed a significant correlation between the combined use of omeprazole, a kind of proton pump inhibitor (PPI), and serum creatinine (Scr) levels with VRC *C*_trough_ in pediatric patients. Despite these insights, these factors cannot fully account for the variability in VRC *C*_trough_. Therefore, further research is needed to identify additional factors influencing VRC *C*_trough_ in pediatric patients.

Recent evidence suggests that inflammation, as measured by C-reactive protein (CRP), is significantly associated with VRC *C*_trough_, identifying it as a novel factor influencing VRC pharmacokinetics in pediatric patients (Valle-T-Figueras et al., 2021). CRP, a widely used marker of inflammation, increases rapidly in response to infection, making it a reliable indicator of the body’s inflammatory status. However, many population pharmacokinetic (PPK) studies have not included CRP levels when predicting VRC *C*_trough_ in pediatric patients (Carlesse et al., 2019; Muto et al., 2015). Our recent systematic review (Hu et al., 2024a) found that, as of 15 August 2023, only four research articles (Valle-T-Figueras et al., 2021; Kang et al., 2020; Luo et al., 2021; Chen et al., 2022) have reported the inflammation as a statistically significant variable influencing VRC *C*_trough_ in children. Further research is needed to better understand the importance of inflammation in VRC dose optimization. Our previous studies on VRC TDM and its clinical applications in pediatric patients did not consider the impact of inflammatory factors. Therefore, we conducted a retrospective study to investigate the factors influencing VRC plasma *C*_trough_ in children, with a particular focus on CRP levels.

## Materials and methods

### Study design

This study was conducted in patients aged ≤18 years at Xiangya Hospital of the Central South University from 1 December 2017, to 31 December 2022. The inclusion criteria were: (i) VRC use for more than 3 days. (ii) Patients who had at least one steady-state VRC *C*_trough_ measurement along with a corresponding CRP concentration obtained on the same day. The exclusion criteria were: (i) Severe liver or kidney dysfunction. (ii) Concurrent use of drugs that significantly affect VRC *C*_trough_, such as P450 enzyme inducers or inhibitors.

### Data collection

Clinical data were collected by reviewing and searching electronic medical records, including VRC dosage, administration routes, concomitant medications, CRP concentration, VRC *C*_trough_, and liver and kidney function indicators. In this study, corticosteroids were the primary treatment for hematologic malignancies, while PPIs were commonly used to prevent gastric mucosal damage associated with chemotherapy or glucocorticoid use (Hu et al., 2023a). Consequently, the study focused on the most frequently prescribed combination therapy for patients with underlying hematologic malignancies: glucocorticoids and PPIs. Albumin (ALB), total bilirubin (TBil), DBil, alanine aminotransferase (ALT), aspartate aminotransferase (AST) are commonly used liver function indicators in clinical practice. BUN and Scr are the most frequently reported renal function indicators associated with VRC *C*_trough_ (Hu et al., 2024a). The estimated glomerular filtration rate (eGFR) was calculated using the modified Schwartz formula. In this study, the normal reference range for CRP was 0–8 mg/L. CRP levels were categorized into three inflammatory groups based on previous studies: mild inflammation (<40 mg/L), moderate inflammation (40–100 mg/L), and severe inflammation (>100 mg/L) (Hu et al., 2024b; Encalada Ventura et al., 2015). Patients were also classified into three age groups: <6, six to <12 and ≥12 years (Boast et al., 2016). Invasive fungal disease (IFD) diagnosis and treatment indications were classified according to the updated EORTC/MSG guidelines (Donnelly et al., 2020).

### Measurement of VRC plasma *C*_trough_

According to the Chinese Pharmaceutical Society (CPS) guidelines (Chen et al., 2018), VRC steady-state *C*_trough_ is measured on day 3 following an oral or intravenous loading dose. In the absence of a loading dose, steady-state *C*_trough_ is measured on days 4–7 of twice-daily dosing. VRC *C*_trough_ measurement was performed using the methods outlined in our previous publication (Hu et al., 2018). Patients may have multiple steady-state VRC *C*_trough_ and CRP concentration measurements taken on the same day during their hospitalization. Consistent with our earlier studies, the therapeutic target range for VRC *C*_trough_ in this study was defined as 1.0–5.5 mg/L (Hu et al., 2018).

### Statistical analysis

All statistical analyses were performed using SPSS version 25.0. Categorical variables are presented as frequencies (percentages), and continuous variables as medians (ranges). To eliminate the influence of dosage, dose-normalized *C*_trough_ values were used in the statistical analysis. Univariate and multivariate analyses were conducted to explore the factors influencing dose-adjusted VRC *C*_trough_ (*C*_trough_/D). In the univariate analysis, continuous variables were assessed using Pearson correlation coefficient, and categorical variables were compared using the *χ* ^2^ test or Fisher’s exact test. No adjustments for multiple comparisons were performed, as this study was an exploratory analysis of factors influencing VRC *C*_trough_.

Multivariate analysis was conducted using a linear mixed model in SPSS to assess the relationship between repeated measurements of *C*_trough_/D and CRP levels. The model was specified as follows:

Y=xβ+Zγ+ξ

Where *Y* represents the response variable (*C*_trough_/D), *x* is the fixed effects design matrix, β is the vector of fixed effect coefficients, Z is the random effects design matrix, γ is the vector of random effect coefficients, and ξ is the residual error term.

Patient ID was included as a random effect to account for repeated measures within individuals. The Wald Type III test was employed to assess the significance of fixed effects after adjusting for the following covariates: sex, body weight, route of administration, age group, concomitant use of PPIs and glucocorticoids, ALB, TBil, DBil, ALT, AST, BUN, and Scr. Model estimates and corresponding 95% confidence intervals (CIs) were used to quantify the strength and direction of associations between covariates and VRC *C*_trough_/D. A two-sided *P* value of <0.05 was considered statistically significant.

## Results

### Characteristics of patients

A total of 59 patients with hematological diseases were included in this study. The median weight was 37.9 kg (range, 10.0–77.7 kg), and males constituted 54.2% (32/59) of the cohort. The median age was 13 years (range, 1–18 years). The most common underlying condition was acute myeloid leukemia (n = 30, 50.8%). Additionally, 30.5% (18/59) of patients had undergone allogeneic hematopoietic stem cell transplantation. A summary of patient characteristics is provided in Table 1.

**TABLE 1:** Patient characteristics.

Characteristics	Total (N = 59)^a^
Demographic Age (yr) Weight (kg) Male, weight (kg) Female, weight (kg)	13 (1-18) 37.9 (10.0-77.7) 32 (54.2), 34.0 (11.0–77.0) 27 (45.8), 34.0 (10.0–60.4)
Underlying disease Acute myeloid leukemia Acute lymphoblastic leukemia Aplastic anemia Myelodysplastic syndrome Lymphoma Thalassemia Others	30 (50.8) 10 (16.9) 7 (11.9) 4 (6.8) 4 (6.8) 2 (3.4) 2 (3.4)
CRP (mg/L)	52.35 (2.41-470.00)
IFD diagnosis Proven Probable Possible	5 (8.5) 22 (37.3) 32 (54.2)
Treatment indication Therapeutic Empirical Prophylactic	21 (35.6) 15 (25.4) 23 (39.0)

^a^ Categorical variables are expressed as number (%) and continuous variables as median (range).

CRP, C-reactive protein. IFD, Invasive fungal disease; yr, years. kg, kilogram.

### VRC use and plasma *C*_trough_

This study included a total of 90 VRC *C*_trough_ measurements from all patients. The median number of VRC *C*_trough_ measurements per patient was 1 (range, 1-10). Of the 59 patients, 76.3% (45/59) received oral VRC, and the median maintenance dose was 5.99 mg/kg (range, 1.35–11.90 mg/kg) administered twice daily. The median VRC *C*_trough_ was 2.60 mg/L (range, 0.01–9.35 mg/L). The proportion of *C*_trough_ within the therapeutic target range was 48.9% (44/90), while 33.3% (30/90) of patients were subtherapeutic and 17.8% (16/90) of patients were supratherapeutic. Additionally, 57.6% (34/59) of the patients received concomitant treatment with PPIs while on VRC, and 42.4% (25/59) used glucocorticoids alongside VRC.

### Factors affecting the VRC *C*_trough_/D by univariate analysis

**FIGURE 1:**
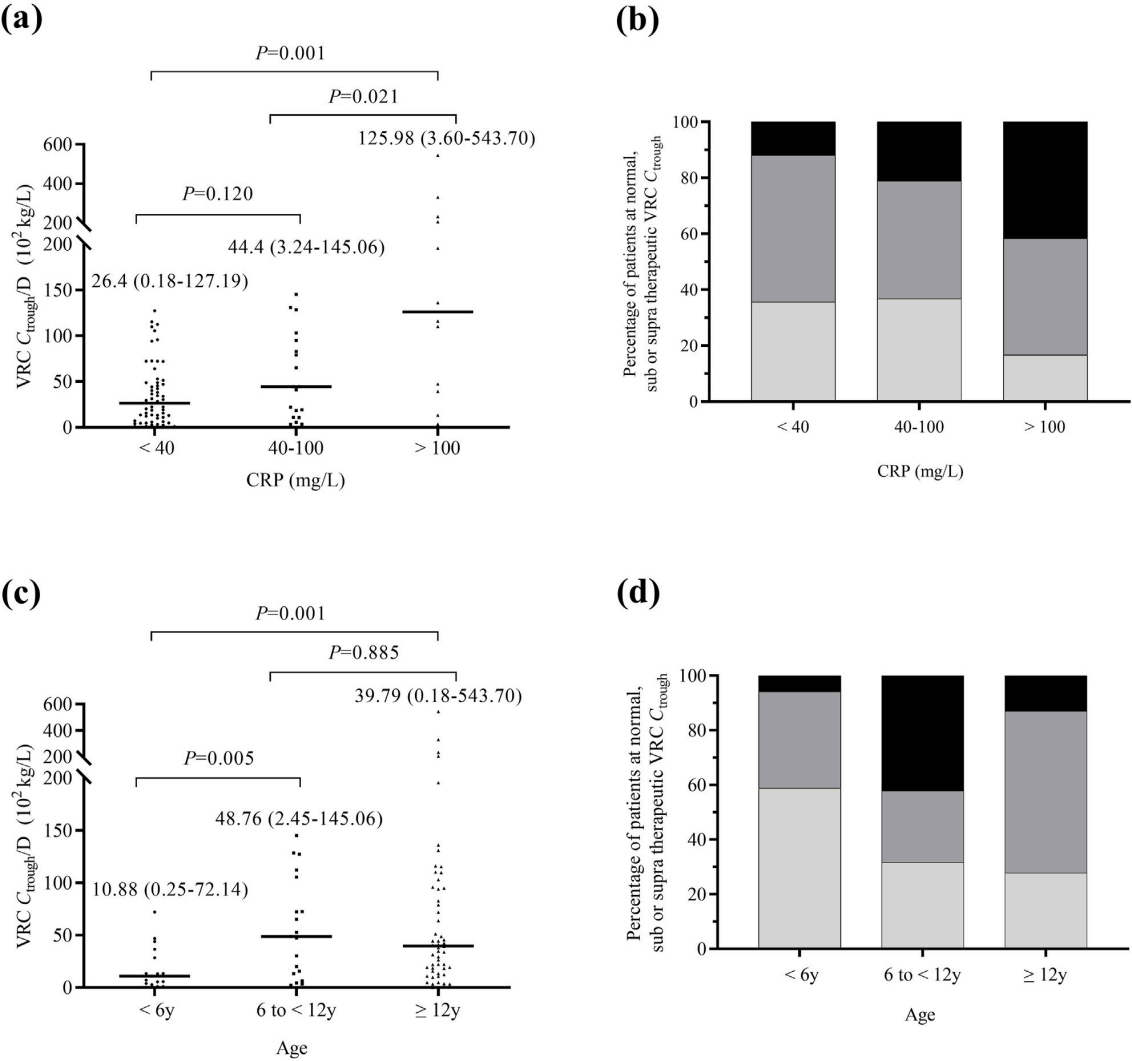
Comparison of VRC *C*_trough_ across different inflammation and age groups. **(a)** Comparison of all measured VRC *C*_trough_/D in patients with mild inflammation (n = 59), moderate inflammation (n = 19), and severe inflammation (n = 12). **(b)** Percentage of patients achieving therapeutic (dark grey), subtherapeutic (light grey), or supratherapeutic (black) VRC *C*_trough_ across different inflammation groups. **(c)** Comparison of VRC *C*_trough_/D in patients aged <6 (n = 17), 6 to <12 (n = 19) and ≥12 (n = 54) years. **(d)** Percentage of patients achieving therapeutic (dark grey), subtherapeutic (light grey), or supratherapeutic (black) VRC *C*_trough_ across different age groups. The *P* value for each group are indicated above the figure. The horizontal bars represent the median values for each group. y, years. VRC, voriconazole. CRP, C-reactive protein. *C*_trough_/D, dose-adjusted trough concentrations.

The Pearson correlation test revealed a significant relationship between VRC *C*_trough_/D and CRP concentrations (n = 90, r = 0.746, *P* < 0.001). In patients with mild (n = 59, 65.6%), moderate (n = 19, 21.1%), and severe (n = 12, 13.3%) inflammation, the median VRC *C*_trough_ were 1.56 mg/L (range, 0.01–9.35 mg/L), 2.08 mg/L (range, 0.13–8.58 mg/L), and 4.40 mg/L (range, 0.12–7.66 mg/L), respectively. Patients with severe inflammation had significantly higher VRC *C*_trough_/D than those with mild inflammation (*P* = 0.001), as shown in Figure 1a. The proportion of supratherapeutic concentrations in the severe inflammation group was the highest, at 41.7%, which was significantly higher than that in the mild inflammation group (41.7% vs. 11.9%, *P* = 0.037), but no significant difference was observed when compared to the moderate inflammation group (41.7% vs. 21.1%, *P* = 0.253), as shown in Figure 1b.

The VRC *C*_trough_/D in patients aged ≥12 years and six to <12 years was significantly higher than in those aged <6 years (*P* = 0.001, *P* = 0.005), as shown in Figure 1c. The percentage of patients with subtherapeutic VRC *C*_trough_ was significantly higher in those aged <6 years compared to patients aged ≥12 years (58.8% vs 27.8%, *P* = 0.019). Figure 1d shows the percentage of patients achieving therapeutic, subtherapeutic, or supratherapeutic VRC *C*_trough_ across different age groups. A negative correlation was observed between ALB and VRC *C*_trough_/D (*P* < 0.001), while TBil, DBil, and Scr were positively correlated with VRC *C*_trough_/D (*P* = 0.015, *P* = 0.006, *P* < 0.001). No significant correlations were found for other factors.

### Multivariate analysis by linear mixed model

The linear mixed model analysis revealed a significant association between VRC *C*_trough_/D and CRP (β = 0.448, 95% CI: 0.309–0.587). Additionally, TBil (*P* = 0.039), DBil (*P* = 0.034), ALB (*P* = 0.011), and Scr (*P* = 0.008) were significantly associated with VRC *C*_trough_/D. No significant associations were found for other covariates, as shown in Table 2.

**TABLE 2:** Multivariate analysis by linear mixed model of the factors affecting the VRC *C*_trough_/D.

Variable	β (95%CI)	*P* Value
Female	3.618 (−15.950, 23.187)	0.714
Weight	0.312 (−0.487, 1.111)	0.440
Intravenous administration^b^	3.991 (−22.508, 30.491)	0.765
Age group^a^ 6 to <12 years ≥12 years	−5.927 (−41.026, 29.171) 35.631 (−4.688, 75.949)	0.738 0.083
CRP	0.448 (0.309, 0.587)	0.000
Concomitant medication PPIs Glucocorticoids	−4.926 (−26.334, 16.482) 0.761 (−22.312, 23.834)	0.649 0.948
Liver function indicators ALB TBil DBil ALT AST	−3.439 (−6.063, −0.815) −6.567 (−12.787, −0.346) 13.050 (1.041, 25.058) −0.240 (−0.608, 0.127) 0.241 (−0.073, 0.555)	0.011 0.039 0.034 0.197 0.131
Renal function indicators BUN Scr	−1.653 (−7.087, 3.782) 1.292 (0.351, 2.232)	0.547 0.008

^a^ Compared to age <6 years.

^b^ Compared to oral administration.

VRC, voriconazole. CRP, C-reactive protein. PPIs, proton pump inhibitors; ALB, albumin; TBil, total bilirubin; DBil, direct bilirubin; ALT, alanine aminotransferase; AST, aspartate aminotransferase; BUN, blood urea nitrogen. Scr, serum creatinine. *C*_trough_/D, dose-adjusted trough concentrations.

### The relationship of VRC *C*_trough_/D and CRP levels in different age groups

**FIGURE 2:**
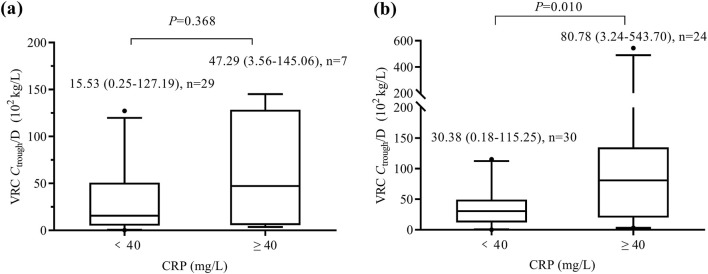
Box and whisker plots of VRC *C*_trough_/D in patients aged <12 years **(a)** and those aged ≥12 years **(b)**. The number of patients and *P* value for each group are indicated above the figure. VRC, voriconazole. CRP, C-reactive protein. *C*_trough_/D, dose-adjusted trough concentrations.

The Pearson correlation test revealed a significant correlation between VRC *C*_trough_/D and CRP concentrations in patients aged ≥12 years (n = 54, r = 0.784, *P* < 0.001), whereas no correlation was found in patients aged <12 years (n = 36, r = 0.199, *P* = 0.244). As shown in Figure 2, no significant difference (*P* = 0.368) in VRC *C*_trough_/D was observed between the mild inflammation group and the moderate-to-severe inflammation group in patients aged <12 years. However, in patients aged ≥12 years, the moderate-to-severe inflammation group exhibited significantly higher VRC *C*_trough_/D than the mild inflammation group (*P* = 0.010).

### Typical cases of VRC-CRP concentrations overtime

**FIGURE 3:**
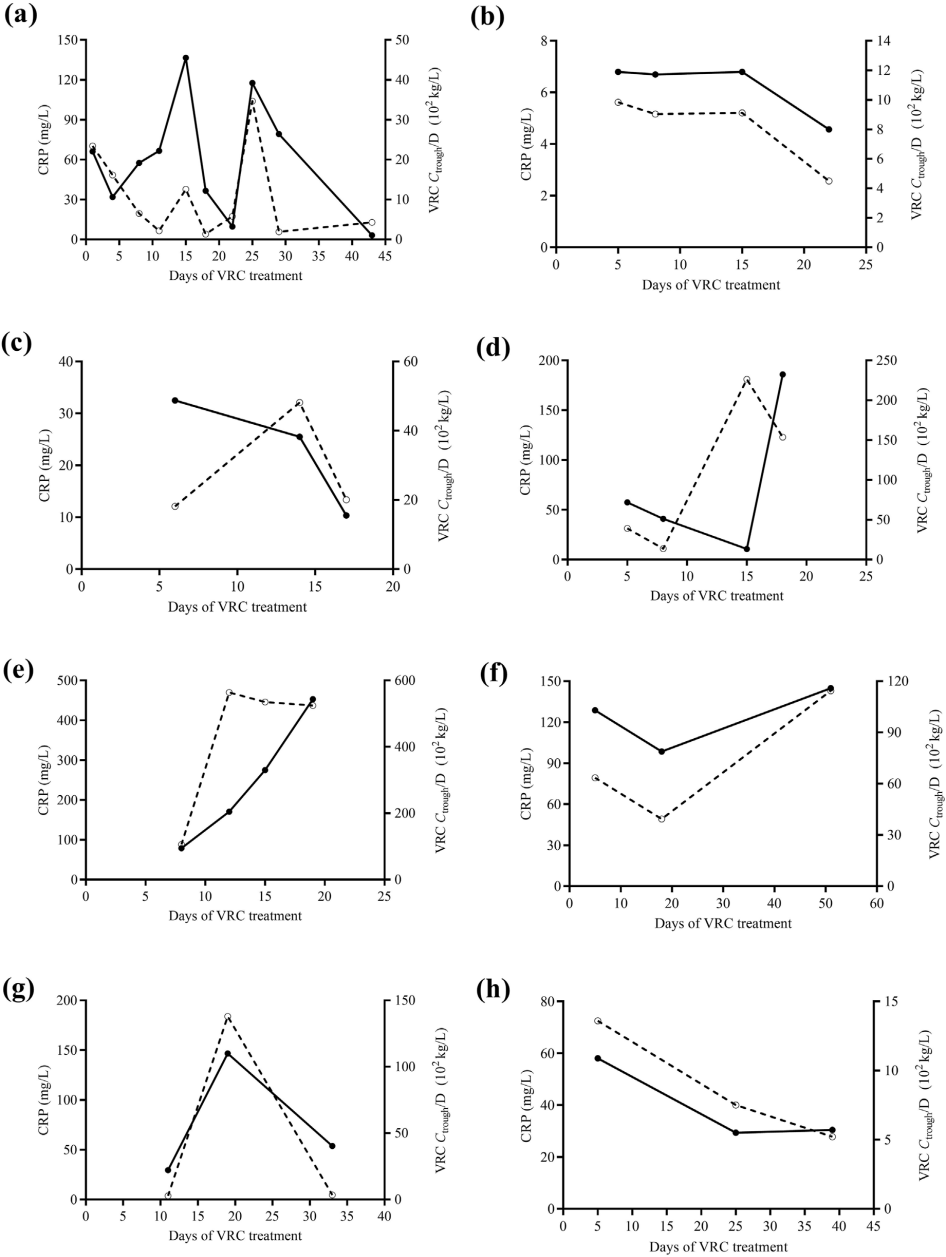
The changes in VRC *C*_trough_/D (solid curve) and CRP levels (dashed curve) over time in eight pediatric patients **(a-h)**, each with at least three VRC *C*_trough_ measurements. VRC, voriconazole. CRP, C-reactive protein. *C*_trough_/D, dose-adjusted trough concentrations.

In this study, eight patients had VRC *C*_trough_ measured ≥3 times. A significant correlation between VRC *C*_trough_/D and CRP concentrations was observed in these eight patients. Figure 3 shows the changes in VRC *C*_trough_/D and CRP levels over time for these eight patients.

## Discussion

This study investigated the impact of non-genetic factors, particularly CRP concentration, on the VRC *C*_trough_ in Chinese pediatric patients. Our retrospective analysis identified that CRP concentration was a key determinant of VRC *C*_trough_, providing essential insights for future dose optimization studies of VRC in children.

VRC is primarily metabolized by the hepatic enzymes CYP2C19, CYP3A4, and CYP3A5. Inflammation triggers the release of cytokines, including tumor necrosis factor-alpha (TNF-α), interleukin-1 (IL-1), and IL-6, which modulate the activity of transcription factors in the liver (Truffot et al., 2018; Aitken et al., 2006). These cytokine-induced changes lead to the downregulation of several CYP genes, resulting in reduced expression of metabolic enzymes and, consequently, a decreased clearance rate of VRC. Studies have shown that different pro-inflammatory cytokines impact various CYP450 enzyme subtypes. Specifically, IL-1, TNF-α, and IL-6 are known to downregulate CYP3A4 activity, while IL-6 predominantly affects CYP2C19 and CYP2C9 activity *in vitro* (Dickmann et al., 2012). However, due to the infrequent clinical monitoring of IL-1, IL-6, and TNF-α, we focused solely on CRP as a representative biomarker for assessing inflammatory status.

CRP is a commonly used clinical marker for assessing the severity of inflammation. Although CRP levels rise at a slower rate compared to IL-1, TNF-α, and IL-6, they start to increase within 4–6 h after the onset of inflammation or infection and typically reach peak levels within 24–48 h (Clyne and Olshaker, 1999). Given the rapid fluctuations in CRP concentrations, we limited our study to patients for whom both VRC *C*_trough_ and CRP levels were measured on the same day to ensure accurate correlation.

Several studies have reported that for every 1.0 mg/L increase in CRP concentration, VRC *C*_trough_ increased by 0.015 mg/L, 0.021 mg/L, or 0.006 mg/L (Luo et al., 2021; van Wanrooy et al., 2014; Aiuchi et al., 2022). Furthermore, Veringa et al. (2017) recommended frequent monitoring of VRC plasma *C*_trough_ during periods of severe inflammation. Similarly, Gautier-Veyret et al. (2019) identified CRP level classification (based on a median CRP threshold of 96 mg/L) as the sole independent risk factor for VRC overdose. In our study, patients did not receive a standardized maintenance dose, and the dosing varied considerably based on body weight. To mitigate the impact of dosage, we used dose normalized *C*_trough_ for analysis. Our study also found that higher VRC *C*_trough_/D were associated with severe inflammation. Previous research has shown that elevated VRC *C*_trough_ are linked to a higher incidence of adverse drug reactions (ADRs) (Jin et al., 2016). Consequently, close monitoring of VRC *C*_trough_ is crucial to prevent ADRs or treatment discontinuation due to excessive concentrations in patients with severe inflammation.

When the patients were divided into different age groups, CRP concentrations were found to be significantly associated with VRC *C*_trough_/D in patients aged ≥12 years, but not in those aged <12 years. This finding is consistent with previous studies that have demonstrated an age-related relationship between VRC *C*_trough_/D and CRP levels in children. For example, Luo et al. (Luo et al., 2021) observed a significant association between CRP levels and VRC pharmacokinetics in patients aged 11–18 years, but not in those aged 2–10 years. In this study, we observed that children aged <6 years had a higher likelihood of achieving subtherapeutic concentrations compared to other age groups. Our previous research also indicated that younger children required higher maintenance doses to reach the therapeutic target range (Hu et al., 2018). This age-related difference may be attributed to variations in the role of CYP2C19, CYP3A4, and flavin-containing monooxygenase 3 (FMO-3) in VRC N-oxidation by liver microsomes between children and adults. Specifically, there are pharmacokinetic differences in VRC between patients aged <12 years and those aged ≥12 years. The clearance rate of VRC in children aged 2–11 years is observed to be nearly three times higher than in adults. Consequently, the metabolic activity of CYP2C19 and FMO-3 may be more pronounced in younger children, leading to a reduced impact of inflammation-induced downregulation of CYP2C19 isoenzymes on VRC metabolism. Additionally, higher liver blood flow and a more pronounced first-pass effect in younger pediatric patients may also contribute to these age-related differences (Yanni et al., 2010). However, the limited sample size in our study may impact the reliability and generalizability of the results. Further research is needed to investigate the physiological reasons behind the lack of correlation between CRP and VRC *C*_trough_ in younger children (<12 years), including enzyme expression and metabolism differences.

Several studies have also reported that other inflammatory markers, such as procalcitonin (PCT), are associated with VRC *C*_trough_. Zeng et al. (Zeng et al., 2020) identified PCT as an independent factor influencing VRC *C*_trough_, suggesting that elevated PCT levels may be linked to an increased risk of ADRs. Similarly, Cheng et al. (Cheng et al., 2020) found that receiver operating characteristic curve analysis indicated that a PCT concentration ≥1.31 ng/mL was associated with a higher incidence of VRC *C*_trough_ > 5 μg/mL. However, due to limited data on PCT testing in pediatric patients in this study, we were unable to include PCT as an inflammatory marker. Future prospective studies should consider PCT concentration as a potential factor influencing VRC *C*_trough_.

In our previous research, we observed a correlation between ALB levels and VRC *C*_trough_ (Hu et al., 2023a). Similarly, Liu et al. (Liu et al., 2017) reported a significant positive correlation between VRC *C*_trough_ and Scr levels in pediatric patients, which aligns with our findings. According to the VRC prescribing information, patients with a creatinine clearance rate <50 mL/min may experience accumulation of the excipient sulfonamide betacyclodextrin sodium when using VRC injection. In such cases, oral formulations are recommended to mitigate this risk (FDA drug label information of voriconazole).

## Limitations

One limitation of this study is the relatively small sample size, as only a limited number of patients met the inclusion criteria of having both VRC *C*_trough_ and CRP concentration measured on the same day. Future studies should aim to increase the sample size to enhance the statistical power and the generalizability of the findings. In this study, eGFR could not be calculated for 30 patients (50.8%) due to missing height data, resulting in its exclusion from the analysis of influencing factors. Additionally, this study did not account for CYP2C19 genetic polymorphisms as a potential factor influencing VRC *C*_trough_. Approximately one-quarter of the patients underwent CYP2C19 genetic testing, which was insufficient to include this factor in the analysis. Aiuchi et al. (Aiuchi et al., 2022) found that the effect of CYP2C19 genotype on VRC metabolism and *C*_trough_ varied across different levels of inflammation, with significant effects observed only in the CRP <40 mg/L group and no significant effects in the CRP ≥40 mg/L group. In our study, 34.4% (31/90) of the patients had CRP ≥40 mg/L, suggesting that the impact of CYP2C19 genotype on VRC *C*_trough_ may not be significant in this cohort. These limitations may constrain the applicability of PPK research in this study. In the future, we aim to expand the sample size and conduct prospective studies incorporating CYP2C19 genotyping, CRP concentrations, and other relevant patient data. It is important to note that our findings are specific to the Chinese population. Due to ethnic differences, the polymorphism of the metabolic enzyme CYP2C19 may vary across populations, which could limit the generalizability of our conclusions. Multiple comparison corrections were not applied to the covariate analyses due to the exploratory nature of this study. Readers are advised to interpret these findings with caution until they are independently validated. Nevertheless, we remain committed to exploring personalized VRC treatment strategies for pediatric patients through ongoing research to ensure the safety and efficacy of VRC in this population.

## Conclusion

Our findings demonstrate a significant association between CRP levels and VRC *C*_trough_, providing an explanation for a portion of the observed variability in VRC exposure. However, the relationship between VRC *C*_trough_ and CRP levels varies across age groups in children, and should be analyzed separately by age. CRP levels may be a key factor influencing VRC *C*_trough_ in pediatric and adolescent populations. Large-scale prospective studies are needed to validate the role of CRP in dose optimization. Additionally, future research should include a more comprehensive analysis that incorporates CYP2C19 polymorphism data to fully elucidate the interplay of genetic and inflammatory factors in VRC pharmacokinetics.

## Data Availability

The original contributions presented in the study are included in the article/supplementary material, further inquiries can be directed to the corresponding author.
